# Gallstone Enteropathy: An Unusual Cause of Bowel Obstruction

**DOI:** 10.7759/cureus.44707

**Published:** 2023-09-05

**Authors:** Venkata Vinod Kumar Matli, Kevin C Marler, Andre Morgan, Varsha Pujala, Sudha Pandit, James Morris

**Affiliations:** 1 Internal Medicine, Christus Highland Medical Center, Shreveport, USA; 2 General Surgery, Christus Highland Medical Center, Shreveport, USA; 3 Gastroenterology and Hepatology, Louisiana State University Health Sciences Center, Shreveport, USA

**Keywords:** biliary ileus, endoscopic lithotripsy, colonic gallstone ileus, gall stone disease, bowel obstruction

## Abstract

Gallstones causing bowel obstruction, known as gallstone ileus, are rare and account for less than 0.5% of small bowel obstruction cases. Additionally, it is a rare complication affecting only 0.3% of patients who have gallstones.

Fistula formation between the biliary system, most commonly between the gallbladder and duodenum because of their proximity, facilitates the migration of gallstones into the enteric system with subsequent impaction in the small intestine, usually in the distal ileum close to the ileocecal valve, promoting the development of mechanical small bowel obstruction.

Computerized tomography of the abdomen and pelvis is a confirmatory and widely used imaging study when there are two signs of Rigler’s triad, which includes pneumobilia, evidence of small bowel obstruction and the presence of radiopaque stones.

We report a case of a 75-year-old Caucasian man who presented with abdominal distention with signs of severe dehydration secondary to intractable nausea and vomiting complicated with severe acute kidney injury and was found to have a 4.7-centimeter gallstone-induced small intestinal obstruction.

## Introduction

Bowel obstruction means impaired flow of the intestinal contents, and it develops secondary to either extraluminal or luminal pathology. Gallstones causing bowel obstruction, known as gallstone ileus, are rare and account for less than 0.5% of mechanical small bowel obstruction (SBO) cases [1]. Additionally, this is a rare complication with a rate of 0.3%-0.5% among patients who have gallstones [2]. SBO is a common acute surgical abdomen, which we often see in our daily practice as internists, accounting for 15% of hospital admissions [3]. We report a case of a 75-year-old Caucasian male who presented with abdominal distention with signs of severe dehydration secondary to intractable nausea and vomiting complicated with severe acute kidney injury and was found to have a 4.7-centimeter gallstone-induced small intestinal obstruction.

## Case presentation

A 75-year-old Caucasian man with a past medical history significant for gastroesophageal reflux disease and essential hypertension was hospitalized for a two-day history of abdominal discomfort with associated intractable nausea; non-bloody, bilious vomiting; and generalized weakness.

His symptoms were continuous, and he was unable to tolerate oral intake. Another pertinent history includes tonsillectomy but no abdominal surgeries. He was hypotensive (blood pressure 74/54 mm hg) and tachycardic at presentation, responded to fluid resuscitation, and became normotensive.

His physical exam was notable for generalized distress and dry mucous membranes with diminished skin turgor. The abdomen was distended and diffusely tender but soft, with high-pitched, tinkling bowel sounds heard over the lower abdominal quadrants.

His complete blood count was significant for leukocytosis and hemoconcentration, as shown in the table. His biochemical profile was significant for mild hyponatremia, hypokalemia, and severe prerenal azotemia as shown in the table and liver function tests and serum lipase levels were within normal limits.

**Table 1:** Laboratory investigations Sr: Serum

Lab parameter	Patient’s values	Reference range
White blood cell count	13,100 cells/microliter (c/mcL)	4000-10,000 c/mcL
Hemoglobin	15.6 grams/deciliter (gms/dL)	13.6-17.0 gms/dL
Hematocrit	45%	41%-50%
Sr sodium	131 milli Equivalents/liter (mEq/L)	135-145 mEq/L
Sr potassium	3.4 mEq/L	3.5-5.5 mEq/L
Sr creatinine	4.6 milligrams/dL (mg/dL)	0.8-1.3 mg/dL
Blood Urea Nitrogen(BUN)	53 mg/dL	7-22 mg/dL
Sr lactate	5.0 millimoles/L (mms/L)	2-4 mms/L

**Figure 1:**
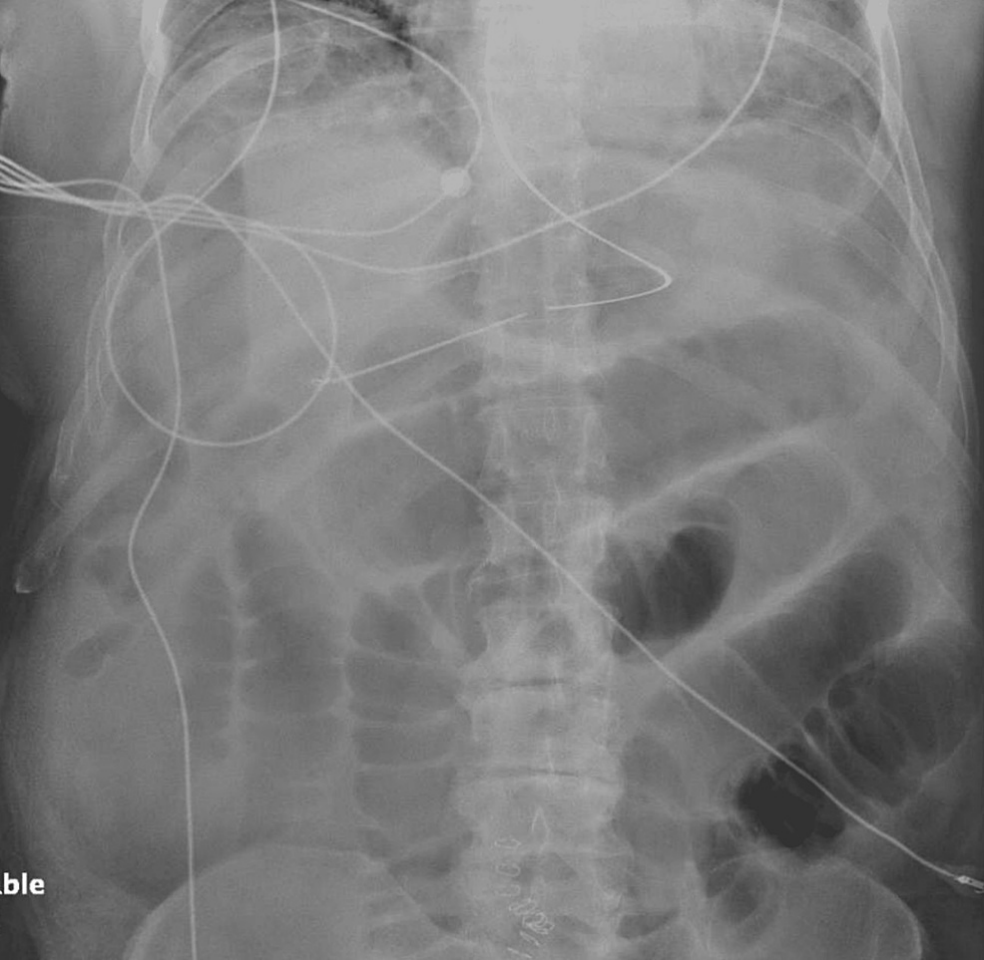
X-ray of the abdomen showing dilated small bowel loops in the mid and upper abdomen with a relative paucity of bowel gas in the colon.

X-ray of the abdomen (Figure 1) showed dilated bowel loops with air-fluid levels with a paucity of colonic gas patterns.

**Figure 2:**
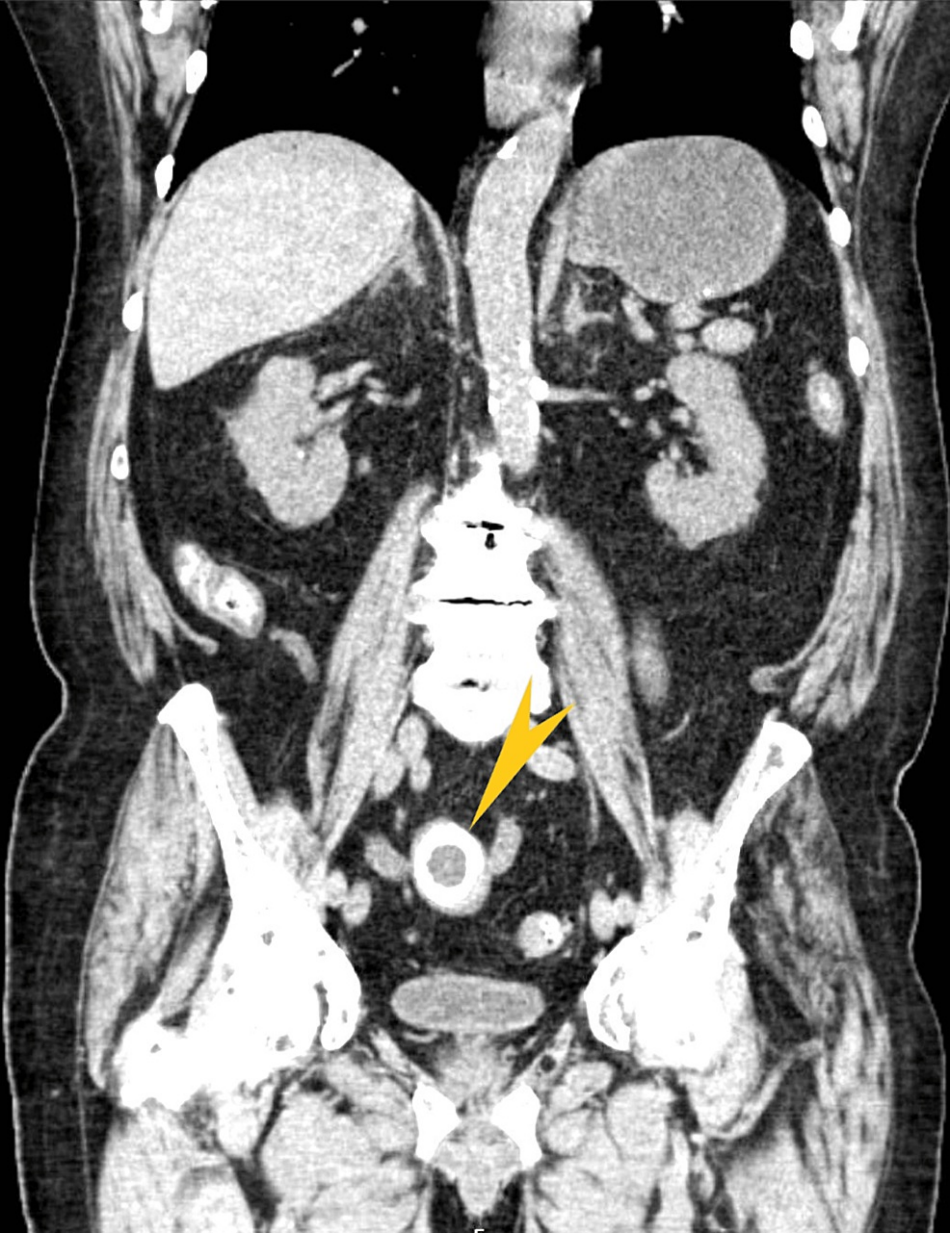
Computerized tomography of the abdomen and pelvis (coronal view) The arrow shows a large, lamellated calcification measuring 4.7 cm and 3.0 cm in the lower midline pelvis.

**Figure 3:**
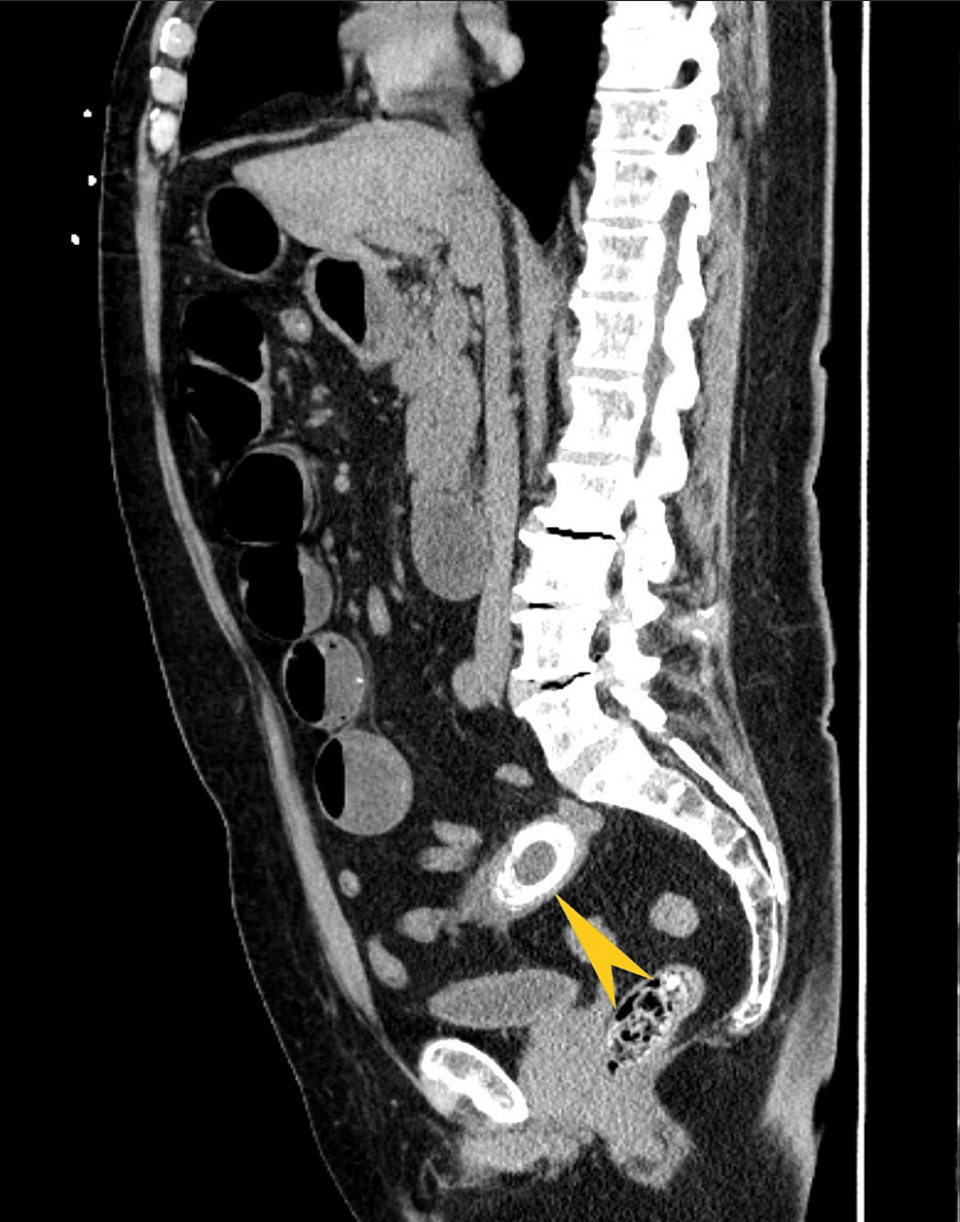
Computerized tomography of the abdomen and pelvis (sagittal view) The arrow shows a large, lamellated calcification measuring 4.7 cm and 3.0 cm in the lower midline pelvis and dilated small bowel loops with air-fluid levels.

**Figure 4:**
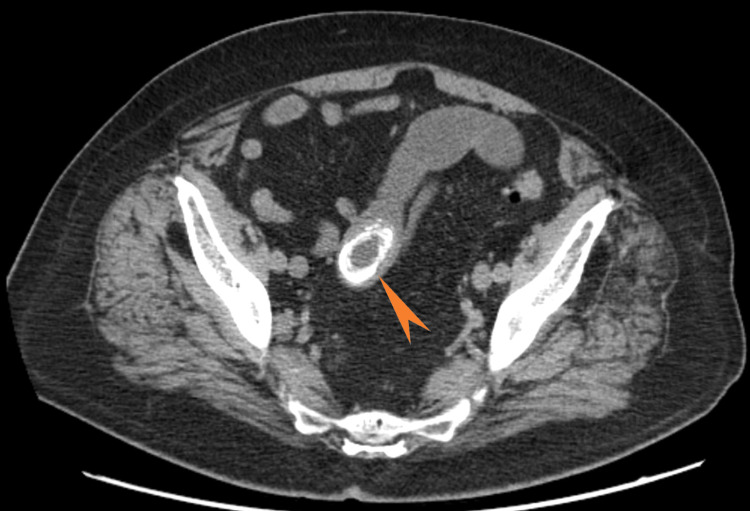
Computerized tomography of the abdomen and pelvis (axial view) The arrow shows large calcifications with dilated small bowel loops.

Computed tomography (CT) of the abdomen and pelvis without contrast showed (Figures 2-4) dilated small bowel loops with a transition point in the lower midline pelvis and a large, lamellated (4.7 cm × 3.0 cm) calcification in the lower midline pelvis.

**Figure 5:**
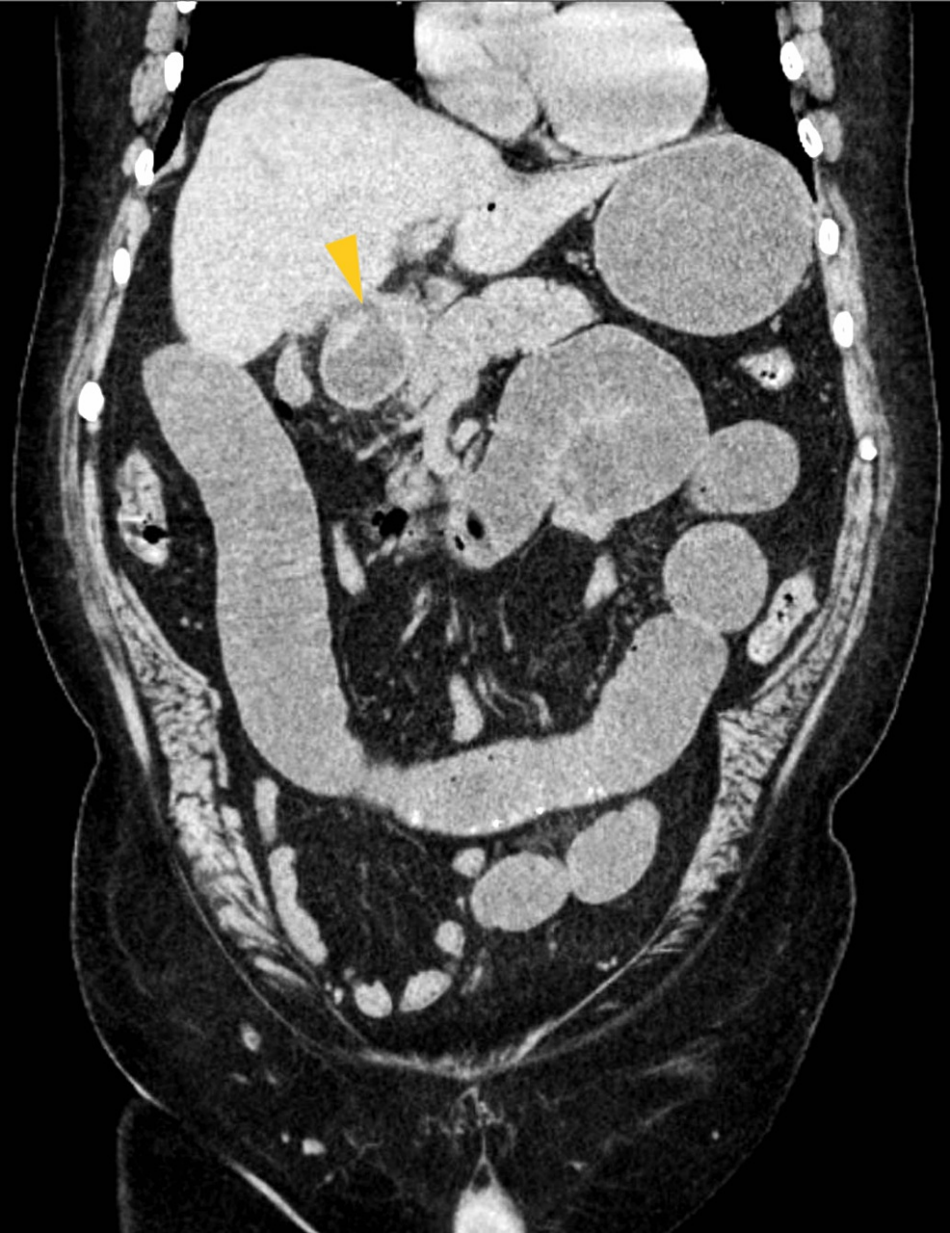
Computerized tomography of abdomen and pelvis (coronal view) The arrow shows the effacement of the fascial planes between the gall bladder and duodenum (yellow pointed triangle) with dilated small bowel loops.

Figure 5 shows effacement of the fascial plane between the duodenum and gallbladder with induration of the gallbladder fossa. These findings were suggestive of gallstone ileus. 

**Figure 6:**
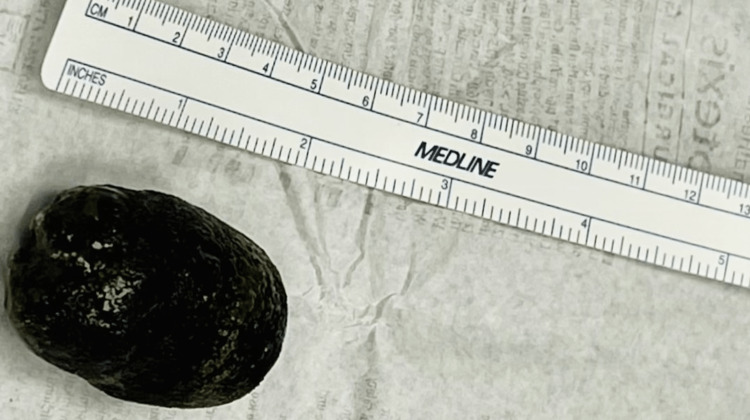
A large dark green gallstone that was milked out during enterotomy Gallstone dimensions: 4.7 cm × 3.0 cm × 2.5 cm

The patient was aggressively treated with nasogastric suction and fluid resuscitation. The patient underwent laparotomy, and the gallstone was easily identified and milked proximally. We found no other stones proximal to this stone. A vertical incision along the small bowel allowed for the removal of a large, dark green-colored gallstone measuring 4.7 cm × 3.0 cm × 2.5 cm (Figure 6), and the enterotomy was closed in a horizontal fashion. His postoperative course was uneventful and required six days of hospital stay. The patient was doing well at a two-week follow-up visit.

## Discussion

Small bowel obstruction secondary to gallstone ileus accounts for less than 0.5% of cases [1]. Intraperitoneal adhesions are the most common cause of SBO, followed by tumors and hernias. Other unusual causes include intussusception (4%) [4], volvulus (4%) [5], and Crohn’s disease (3%) [6].

Fistula formation is an important process for the facilitation of gallstone migration into the enteric system. Chronic inflammation of the gallbladder causes adhesiveness between it and the duodenum; over a period, stone pressure causes necrosis of the gallbladder wall, resulting in fistula formation, which is most commonly cholecystoduodenal because of the duodenal proximity to the gallbladder. Cholecystocolonic and cholecystogastric are other possibilities [7]. Stone migration causes episodic obstruction, and the usual site of impaction is the ileum close to the ileocecal valve, which is the narrowest part of the small intestine. It can become impacted in the duodenum, causing gastric outlet obstruction called Bouveret’s syndrome, which is seen in 1-3% of patients with gallstone ileus [8]. Obstructive symptoms are episodic initially as the stone wanders through the intestine and then becomes permanent after it lodges in the narrowest part of the intestine.

The biochemical profile is nonspecific, and imaging studies are diagnostic. X-ray abdomen upright shows classic findings, including dilated loops of the small bowel with air-fluid levels, pneumobilia (air in the biliary tree), and a radiopaque gallstone in the right iliac fossa, called Rigler’s triad. To confirm the diagnosis, the patient needs contrast-enhanced CT of the abdomen and pelvis, which is a widely used imaging study and needs to have at least two of the three signs of the Rigler’s triad. Pneumobilia is not a signature sign of gallstone ileus; it can be seen after endoscopic biliary procedures involving sphincterotomy, sphincter of Oddi dysfunction, and postoperatively in cholecystectomy patients. Lassandro et al. evaluated various imaging studies in 27 gallstone bowel obstruction patients in a retrospective study comparing abdominal X-ray, abdominal ultrasound, and contrast-enhanced CT of the abdomen and pelvis. CT has the highest sensitivity for confirming signs of bowel obstruction at 96.3%, followed by pneumobilia at 88.9% and gallstones at 81.8% [9].

The risk of bowel obstruction lies in the size of the stone. El-Amin et al. studied patients with double contrast barium enema examination and colonoscopy and showed that the mean ileocecal valve height was 1.7 cm and the mean width was 2.8 cm [10]. Studies have also shown that the stone needs to be at least 2 cm in diameter to cause an obstruction [11,12].

The first step in the management of gallstone ileus is fluid resuscitation and relieving the intestinal obstruction surgically by enterolithotomy (enterotomy with stone extraction). The next step is the closure of the fistulous communication and cholecystectomy. To date, no consensus has been reached regarding whether all of these procedures can be performed at one time. However, our study and a literature review show that enterolithotomy alone is safe and associated with better outcomes than a one-stage procedure, which should be offered only selectively to patients who have good cardiorespiratory reserve [13,14]. Reisner and Cohen published a review of 1001 reported gallstone ileus cases where they showed that the one-stage procedure carries an associated mortality of 16.9% compared to 11.7% for enterolithotomy alone [15]. Endoscopic lithotripsy should be reserved selectively, such as for Bouverets syndrome and colonic obstruction secondary to gallstones [8]. Zielinski et al. reported a successful colonoscopic electrohydraulic lithotripsy in patients diagnosed with colonic gallstone ileus [16]. The therapeutic option of endoscopy should only be performed by gastroenterologists with advanced endoscopic expertise, and more studies are needed to determine how to use it appropriately and adequately.

## Conclusions

Gallstones causing bowel obstruction, known as gallstone ileus, are rare and account for less than 0.5% of small bowel obstruction cases. Additionally, it is a rare complication affecting less than 0.3% of patients who have gallstones. Contrast-enhanced CT of the abdomen and pelvis is a confirmatory imaging study. This condition should be managed with enterolithotomy alone, which is safer than a one-step procedure including fistula closure and cholecystectomy. Endoscopic lithotripsy can only be performed in select patients and only by endoscopists with advanced expertise. However, more research and studies are required to use lithotripsy appropriately and adequately for this condition.
